# Predicting Occlusion Myocardial Infarctions in the Emergency Department Using Artificial Intelligence

**DOI:** 10.1016/j.acepjo.2025.100299

**Published:** 2026-01-09

**Authors:** Axel Nyström, Anders Björkelund, Henrik Wagner, Ulf Ekelund, Mattias Ohlsson, Jonas Björk, Arash Mokhtari, Jakob Lundager Forberg

**Affiliations:** 1 Department of Laboratory Medicine, Lund University, Lund, Sweden; 2 Computational Science for Health and Environment (COSHE), Centre for Environmental and Climate Science, Lund University, Lund, Sweden; 3 Lund University, Skåne University Hospital, Department of Cardiology, Lund, Sweden; 4 Department of Emergency and Internal Medicine, Skåne University Hospital, Lund, Sweden; 5 Department of Clinical Sciences, Lund University, Lund, Sweden; 6 Center for Applied Intelligent Systems Research (CAISR), Halmstad University, Halmstad, Sweden; 7 Clinical Studies Sweden, Forum South, Skåne University Hospital, Lund, Sweden; 8 Department of Emergency Medicine, Helsingborg Hospital, Helsingborg, Sweden

**Keywords:** artificial intelligence, occlusion myocardial infarction, ECG

## Abstract

**Objectives:**

The objective was to develop an artificial intelligence (AI) model for predicting acute coronary occlusion myocardial infarction (OMI) in patients with chest pain at the emergency department (ED), using information that is widely available early in the ED assessment.

**Methods:**

In a cohort of 24,511 consecutive adult ED patients with chest pain from 5 Swedish hospitals, OMI cases were identified through register data and manual review of health records and angiographies. Ambulance patients bypassing the ED due to ST-elevation myocardial infarction (STEMI) were not included in the cohort. A deep-learning AI model was created to predict OMI using the electrocardiogram, optionally combined with other early ED data, including medical history and initial lab values. The model was internally validated on held-out data and compared with the STEMI criteria.

**Results:**

A total of 467 patients (1.9%) were identified as OMI, corresponding to 29% of all acute myocardial infarction cases. The 30-day mortality rate was 6.6% for OMI, compared with 3.3% for non-OMI. Only 5.4% of the OMI cases received angiography within the guideline-recommended maximum of 90 minutes after ED arrival. The AI model achieved an area under the receiver operating characteristic (AUC) of 95.3% (95% CI, 93.8%-97.3%), with a sensitivity of 62% compared with 27% for the STEMI criteria (difference 34.5%; 95% CI, 22.9%-45.2%) at the same specificity (97.4%).

**Conclusion:**

Our AI model identified OMI in ED patients with chest pain with an AUC of 95%, doubling sensitivity compared with the STEMI criteria at the same specificity. Using the model could reduce time to intervention, as only about 1 in 20 OMI cases currently receive timely angiography.

**The Bottom Line**

In a cohort of 24,511 patients presenting with chest pain to Swedish emergency departments, we retrospectively identified 467 cases of acute coronary occlusion myocardial infarction (OMI), corresponding to 29% of all myocardial infarctions. Of these, only 5.4% received treatment within the guideline-recommended 90 minutes. We constructed an artificial intelligence model for predicting OMI using the electrocardiogram, initial lab values, and other clinical data available early in the emergency department workflow. The model achieved an area under the receiver operating characteristic of 95% and substantially outperformed standard electrocardiogram ST-elevation criteria, with a doubled sensitivity at the same specificity.

## 1 Introduction

### 1.1 Background

It is well established that acute percutaneous coronary intervention (PCI) is an effective treatment for patients with acute coronary occlusion (ACO),^1^ with guidelines emphasizing the importance of early identification of ACO in order to reduce myocardial ischemic time.^2^, ^3^, ^4^ Consequently, prehospital screening for suspected ACO has been widely implemented to facilitate direct transport to PCI facilities. Current screening methods rely on criteria for ST-segment elevation on the electrocardiogram (ECG), corresponding to ST elevation myocardial infarction (STEMI).^2^^,^^5^

However, growing evidence suggests that STEMI criteria are insufficient for diagnosing ACO, leading to inadequate treatment for many patients. As many as 40% of patients with ACO do not meet STEMI criteria,^6^ which has been linked to a 50% higher case fatality rate, likely due to delays in intervention.^7^ Further, many patients who meet STEMI criteria have no ACO, with one study showing that 36% of STEMI activations in the ED did not represent ACO.^8^ For these reasons, there have been calls for a paradigm shift to replace STEMI with the concept of ACO myocardial infarction (OMI).^9^^,^^10^ The argument is that we should not provide urgent PCI based on the imperfect STEMI criteria but instead focus on the true target of the PCI, ie, a possible ACO. Several attempts have been made to formulate alternative ECG criteria for OMI, but their complexity limits the usefulness for manual interpretation, especially under stressful conditions.^11^, ^12^, ^13^ In this context, artificial intelligence (AI) models have the potential to enhance OMI detection by presenting timely predictions to healthcare professionals.^4^^,^^14^

Efforts to develop AI prediction models for OMI are ongoing and promising, with some already indicating improved performance compared with STEMI criteria on certain populations.^15^^,^^16^

### 1.2 Importance

The potential clinical impact of early direct OMI detection is considerable. A logical next step is to evaluate such models in ED populations where ambulance-identified STEMI patients bypass the ED and go directly to the catheterization lab. In these EDs, OMI cases are more likely to present with subtle ECG changes, making diagnosis particularly challenging. An AI model for OMI detection tailored to this population could thus be a valuable decision-support tool. Furthermore, previously published AI models for OMI have been trained exclusively on the ECG, so the benefit of additional clinical information is still unclear.

### 1.3 Goals of This Investigation

The primary objective of this European multicenter study was to develop and validate an AI model predicting OMI in an ED population where prehospital bypass of STEMI patients is routine. The goal was a model that could utilize different information that becomes available early in the ED process: patient medical history, the initial ECG, point-of-care (POC) blood samples, and the first high-sensitivity cardiac troponin T (hs-cTnT) measurement. We compared our results with the established STEMI criteria for different combinations of model inputs.

## 2 Methods

### 2.1 Study Design and Setting

This was a retrospective study including 24,511 consecutive ED patients with chest pain from the European Society of Cardiology 0-h/1-h Troponin Rule-Out Protocol (ESC-TROP) trial.^17^^,^^18^ The data were collected from 5 hospitals in southern Sweden between Feb 1, 2017, and Nov 30, 2018, and included a 5-year medical history of diagnoses for each patient, treatments, and data from the SWEDEHEART national quality register and the Swedish patient register,^19^ ECGs, lab values, and diagnoses from the ED visit, and a 1-year follow-up of diagnoses. All adult (≥18 years) patients with a primary complaint of nontraumatic chest pain were included by default, with the possibility to opt out at any time for any reason.

**Figure 1:**
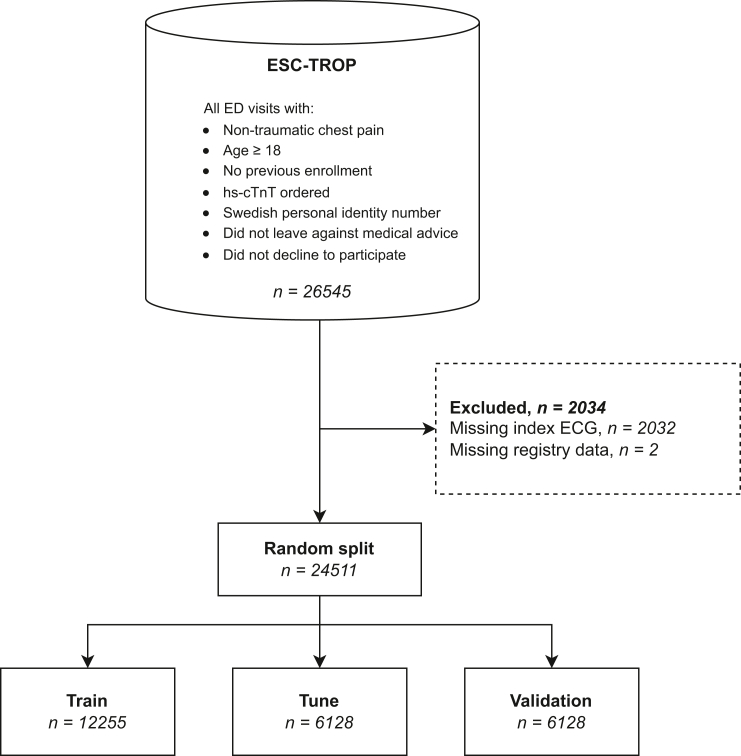
Exclusion criteria and data split. Exclusion criteria and data split. Each patient is only represented once. The random split was stratified on the outcome (OMI), so that each set contained the same proportion of OMI cases. ECG, electrocardiogram; ED, emergency department; hs-cTnT, high-sensitivity cardiac troponin T; OMI, occlusion myocardial infarction.

Exclusion criteria for the ESC-TROP study were previous enrollment, leaving against medical advice, lack of Swedish personal identity number, lack of ordered hs-cTnT, or actively declining to participate. Patients with a STEMI diagnosis during the index visit were excluded from the original ESC-TROP trial, but in the present study, they were kept to more accurately reflect normal patient flow. Conversely, this study excluded patients without ECGs or where the index ECG was of poor technical quality, as well as patients where the OMI outcome was unclear due to missing data, whereas the ESC-TROP study did not. The exclusion criteria used in the present study are visualized in Figure 1.

Importantly, in our setting, patients with chest pain identified as STEMI by an ECG in the ambulance are sent directly to the catheterization lab, bypassing the ED, and are consequently not included in this study. The study was approved by the Regional Ethics Review Board in Lund, Sweden (Dnr 2017/831 and 2018/708). The study received ethical approval without the need for written informed consent.

### 2.2 The Annotation Process

For the clinical outcome, OMI cases were identified through a structured, multistep annotation process. Initially, all patients in the study cohort with a discharge diagnosis of acute myocardial infarction (AMI) and a registered ACO in the Swedish Coronary Angiography and Angioplasty Registry (SCAAR) at the index visit were classified as an OMI case. Those with hs-cTnT levels exceeding 1000 ng/L and who underwent urgent PCI or coronary artery bypass grafting (CABG) were also classified as OMI.

Further, an emergency physician (JLF) reviewed all additional coronary angiography reports from AMI patients and classified as OMI those with acute culprit lesions and thrombolysis in myocardial infarction (TIMI) flow of 0 or 1. In cases with reduced flow but without a clear TIMI classification, a cardiologist (AM) and an emergency physician (JLF) jointly determined the presence of TIMI flow 0 or 1 for OMI classification. Ambiguous cases were further assessed by an invasive cardiologist (HW), who reviewed angiogram recordings to identify OMI cases with acute culprit lesions and TIMI flow 0 or 1.

**Figure 2:**
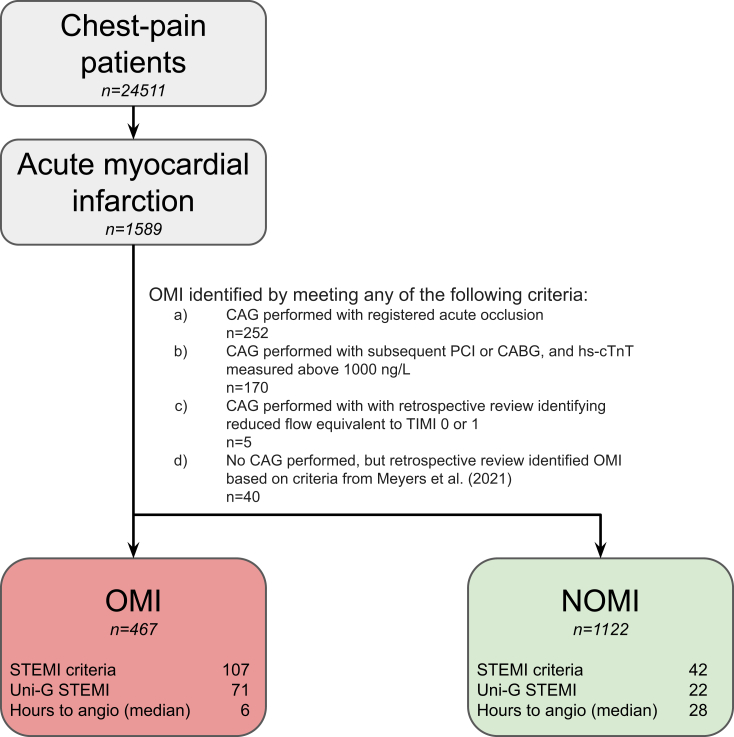
Summary of the occlusion myocardial infarction (OMI) annotation process. Overview of the OMI annotation process. Uni-G STEMI refers to “STEMI equivalent” diagnoses from the University of Glasgow ECG analysis program by MacFarlane et al.^20^ CABG, coronary artery bypass graft surgery; CAG, coronary angiography; hs-cTnT, high-sensitivity cardiac troponin T; NOMI, negative occlusion myocardial infarction; OMI, occlusion myocardial infarction; PCI, percutaneous coronary intervention; STEMI, ST-segment elevation myocardial infarction; TIMI, thrombolysis in myocardial infarction.

For AMI patients who did not undergo angiography, the criteria from Meyers et al^12^ were applied, in which an OMI classification was assigned if hs-cTnT levels exceeded 1000 ng/L and were accompanied by either new or presumed new regional wall motion abnormalities on echocardiography or an ECG indicative of STEMI in patients who experienced cardiac arrest before angiography could be performed. The annotation process is summarized in Figure 2.^20^

Patients undergoing PCI or CABG within 1 week of the ED index visit were identified in the Swedish national patient register and the SCAAR register. For the hs-cTnT variable, we used the maximum hs-cTnT measurement recorded within 24 hours, as well as the hs-cTnT reported to the SWEDEHEART register. It should be noted that while we used the highest hs-cTnT value available, the study hospitals did not routinely monitor hs-cTnT until the peak, so the recorded maximum may not be the actual peak.

### 2.3 The AI Model

The AI model was developed using a supervised machine learning approach. The data were randomly partitioned into a training set (50%), tuning set (25%), and validation set (25%), as indicated by Figure 1. The partitioning was done so that each set contained the same proportion of OMI, and each patient was only represented in one of the sets. The training set was used to derive the model parameters, and the tuning set was used to adjust hyperparameters. The validation set was only used for the final model evaluation, and not for training or tuning.

We divided the model inputs into 4 feature groups, based on when they would become available at the ED in clinical practice. The first group corresponded to the patient’s medical history and included a selection of diagnoses, medications, and treatments from the past 5 years, as well as the patient’s age and sex (male or female, as recorded in the national patient register). The second feature group came from the initial 12-lead 10 s ECG signal, which is usually available within a few minutes after ED arrival. The third group was a selection of quickly available POC blood tests: hemoglobin, creatinine, and glucose. Finally, the fourth group included the initial hs-cTnT measurement (Elecsys assay on Cobas instruments, Roche Diagnostics), where the results are available within 45 minutes. The features are described in more detail in Supplementary Appendix 1.

**Figure 3:**
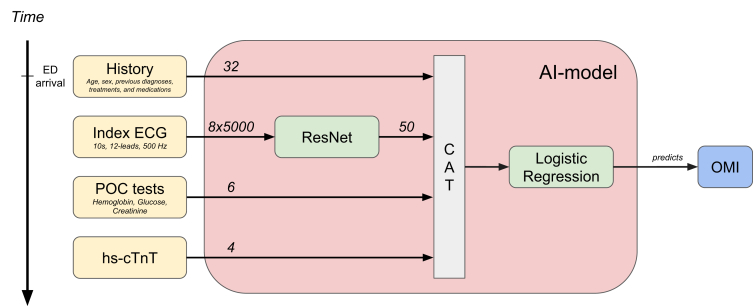
Model structure. The boxes on the left are the feature groups, with the numbers on each arrow indicating their size and shape. The arrow on the left indicates the relative time at which the different feature groups are typically available at the emergency department (ED). POC refers to point-of-care blood samples. CAT means concatenation. Only the 8 linearly independent electrocardiogram (ECG) leads were used as input to the ResNet model. AI, artificial intelligence; CAT, concatenation; ED, emergency department; hs-cTnT, high-sensitivity cardiac troponin T; OMI, occlusion myocardial infarction.

The granularity of the raw ECG signal makes it difficult to combine effectively with the other features. To address this, we created a deep-learning model using the ResNet architecture^21^ to automatically extract a compact 50-dimensional feature representation from each ECG. This ECG representation was subsequently concatenated with the other feature groups, before feeding into a logistic regression model making the final prediction. In order to assess the usefulness of each feature group, we trained and evaluated the model on all possible combinations of inputs. The high-level model structure is illustrated in Figure 3.

#### 2.3.1 The ResNet ECG model

The ResNet architecture, which originates from the field of image recognition, has achieved state-of-the-art results in several ECG analysis tasks.^22^, ^23^, ^24^ The model in this study is a continuation of our previous work, where transfer learning was used to increase classification performance for AMI.^25^ We also employed a number of techniques from the recent literature to further improve the model, as described below.

Following Strodthoff et al,^26^ the ECG signal was randomly cropped to a 2.5 s segment during training. During evaluation, a prediction was made for 10 overlapping 2.5 s segments and the results were averaged. This process functions as a form of regularization, which prevents overfitting.

Following Gustafsson et al,^23^ in cases where a patient has more than 1 ECG recorded during an ED visit, we augmented the training set to include these additional ECG recordings. For each such additional ECG, we used the same outcome label as the index ECG. This increased the number of ECGs in the training set by about 50%, improving the performance of the ECG preprocessing model. The augmentation was only used for training the ResNet model and not during feature extraction at test time.

To reduce the dimensionality of the input signal, only the 8 leads V1-V6, I, and II were used by the ResNet model, because leads III, aVR, aVL, and aVF are linear combinations of leads I and II, and thus do not carry any additional information.

Further details on model selection and tuning are described in Supplementary Appendix 2. All models and analyses were done in Python 3.11 (Python Software Foundation). The ResNet models were implemented using the PyTorch 2.0 library,^27^ running on Nvidia 2080Ti, 3090, and 4090 GPUs.

#### 2.3.2 Logistic regression

All scalar features were normalized in a preprocessing step prior to being fed to the logistic regression model. Specifically, the hs-cTnT and POC blood test features were normalized using the Yeo-Johnson power transformer.^28^ The remaining scalar features were scaled to zero mean and unit variance. The POC blood tests had a missingness between 0.8% (hs-cTnT) and 5% (glucose), primarily due to hemolysis. The indicator variables had a correlation to OMI between 0.004 and 0.019. This suggests that the measurements were missing at random with respect to the OMI outcome, and we therefore imputed the missing values using the corresponding averages on the training set. The parameters for preprocessing were determined from the training set to prevent data leakage. Logistic regression and preprocessing were implemented using the Scikit-learn 1.3 library.^29^

### 2.4 Outcomes

The clinical outcome of this study was OMI. We compared the predictive performance of the AI model at identifying OMI for different combinations of input feature groups, with the STEMI criteria.^2^^,^^5^ We used the rule-based, proprietary Uni-G ECG analysis program to automatically calculate ECG amplitudes and intervals necessary for the STEMI criteria.^20^ The primary evaluation metrics were sensitivity, specificity, positive predictive value (PPV), negative predictive value (NPV), and the area under the receiver operating characteristic curve (AUC), although the latter is only meaningful for nonbinary predictions (ie, not for the STEMI criteria). We used bootstrapping on the validation set to approximate 95% CI for all metrics, with *B* = 1000 bootstrapping samples. For direct comparisons, bootstrapped differences were calculated.

In an additional comparison, we considered certain automated ECG statements from the Uni-G program as STEMI equivalents and evaluated them as predictions of OMI. The statements used were those ending with “ST elevation, CONSIDER ACUTE INFARCT.”

The AI model and the Uni-G STEMI equivalents were primarily evaluated on the full validation set, whereas the STEMI criteria were evaluated only on patients without left bundle branch block (LBBB), left ventricular hypertrophy (LVH), or ventricular pacing (VP), because the criteria are not defined for those patients.^2^^,^^5^ Because this reduced cohort is likely less diagnostically challenging, we additionally evaluated the AI model and the Uni-G STEMI equivalent on the reduced cohort, as a secondary point of comparison.

To simplify the comparison of the AI model to the STEMI criteria in terms of sensitivity and specificity, we selected the decision threshold of the AI model to match the specificity of the STEMI criteria on the tuning set.

## 3 Results

### 3.1 Annotation Results

Out of the 24511 ED patients with chest pain, we identified 467 cases of OMI, which correspond to 1.9% of patients with chest pain, and 29% of AMI cases. Patient characteristics are shown in Table 1. The OMI patients were predominantly male (75%) with a lower prevalence of comorbidities, such as diabetes and prior cardiovascular diseases, compared with OMI-negative AMI (NOMI) patients. The 30-day mortality of OMI patients was roughly twice that of NOMI patients (6.6% vs 3.3%). A total of 613 patients fulfilled the STEMI criteria on the initial ECG, 149 of whom received an AMI diagnosis. Of these, 107 were classified as OMI and 42 as NOMI. Patient characteristics were similar across the training, tuning, and validation splits, as shown in Supplementary Appendix 3: Table S6.

**Table 1:** Patient Characteristics.

	All	OMI	NOMI
Patients, *n*	24511	467	1122
Female, *n* (%)	11,802 (48.1)	119 (25.5)	418 (37.3)
Age, y (std)	59 (18.7)	68 (13.4)	71 (12.9)
AMI, *n* (%)	1589 (6.5)	467 (100.0)	1122 (100.0)
OMI, *n* (%)	467 (1.9)	467 (100.0)	0 (0.0)
NOMI, *n* (%)	1122 (4.6)	0 (0.0)	1122 (100.0)
STEMI criteria, *n* (%)	613 (2.5)	107 (22.9)	42 (3.7)
Ischemic heart diseases, *n* (%)	3624 (14.8)	66 (14.1)	277 (24.7)
Acute myocardial infarction, *n* (%)	1491 (6.1)	34 (7.3)	158 (14.1)
Angina pectoris, *n* (%)	1587 (6.5)	25 (5.4)	125 (11.1)
Unstable angina, *n* (%)	546 (2.2)	14 (3.0)	39 (3.5)
Heart failure, *n* (%)	1456 (5.9)	17 (3.6)	91 (8.1)
Diabetes, *n* (%)	2086 (8.5)	42 (9.0)	163 (14.5)
Hypertension, *n* (%)	5015 (20.5)	93 (19.9)	330 (29.4)
Pulmonary embolism, *n* (%)	302 (1.2)	1 (0.2)	14 (1.2)
Cerebrovascular diseases, *n* (%)	890 (3.6)	15 (3.2)	53 (4.7)
Chronic obstructive pulmonary disease, *n* (%)	925 (3.8)	9 (1.9)	40 (3.6)
Prior CABG, *n* (%)	252 (1.0)	4 (0.9)	21 (1.9)
Prior PCI, *n* (%)	1417 (5.8)	31 (6.6)	114 (10.2)
Antithrombotics, *n* (%)	5721 (23.3)	116 (24.8)	467 (41.6)
Statin, *n* (%)	6685 (27.3)	120 (25.7)	452 (40.3)
Other lipid-lowering medication, *n* (%)	652 (2.7)	11 (2.4)	51 (4.5)
Antihypertensive medication, *n* (%)	8968 (36.6)	196 (42.0)	621 (55.3)
Beta blockers, *n* (%)	7373 (30.1)	139 (29.8)	474 (42.2)
Anticoagulants, *n* (%)	1321 (5.4)	16 (3.4)	57 (5.1)
Insulin, *n* (%)	1393 (5.7)	41 (8.8)	136 (12.1)
Other antidiabetics, *n* (%)	2418 (9.9)	61 (13.1)	190 (16.9)
Diuretics, *n* (%)	3621 (14.8)	60 (12.8)	240 (21.4)
Thiazide, *n* (%)	1115 (4.5)	22 (4.7)	72 (6.4)
ACE/AII-antagonists, *n* (%)	7625 (31.1)	161 (34.5)	539 (48.0)
Initial hs-cTnT (ng/L), median (IQR)	7 (4-15)	105 (29-441)	51 (24-120)
Initial lactate (mmol/L), median (IQR)	1.2 (1.0-1.6)	1.7 (1.2-2.3)	1.4 (1.1-1.9)
30-d mortality, *n* (%)	284 (1.2)	31 (6.6)	37 (3.3)
180-d mortality, *n* (%)	707 (2.9)	44 (9.4)	71 (6.3)
1-y mortality, *n* (%)	1013 (4.1)	48 (10.3)	95 (8.5)
Hours until angiography, median (IQR)	29 (17-60)	6 (3-24)	28 (19-53)
Angiography within 90 min, *n* (%)	32 (0.1)	25 (5.4)	3 (0.3)
Angiography within 2 h, *n* (%)	65 (0.3)	50 (10.7)	11 (1.0)
Angiography within 3 h, *n* (%)	175 (0.7)	133 (28.5)	27 (2.4)
Angiography within 6 h, *n* (%)	321 (1.3)	210 (45.0)	69 (6.1)

ACE, angiotensin-converting enzyme; AII, angiotensin II; AMI, acute myocardial infarction; ATC, anatomical therapeutic chemical; CABG, coronary artery bypass graft; ECG, electrocardiogram; ICD, International Classification of Diseases; NOMI, negative occlusion myocardial infarction; OMI, occlusion myocardial infarction; PCI, percutaneous coronary intervention; STEMI, ST-segment elevation myocardial infarction.

Disease history covers 5 years, medication history 3 years. STEMI criteria were evaluated on the index ECG, treating left bundle branch block, left ventricular hypertrophy, and ventricular pacing as STEMI negative. See Supplementary Appendix 1 for ICD-10 codes and ATC codes corresponding to disease and medication history.

### 3.2 Time Until Angiography

In the OMI cases where angiography was performed (426 out of 467; 91%), the median time until angiography was just under 6½ hours, compared with 28 hours for NOMI patients. Only 25 OMI patients (5.4%) received angiography within the recommended maximum of 90 minutes, compared with 3 (0.3%) for the NOMI group. Four patients received angiography within 90 minutes without an AMI diagnosis.

### 3.3 Machine Learning Results

The validation results of the AI model using cumulative inputs are summarized in Table 2, together with the automated evaluation of the STEMI criteria and the ECG-based STEMI interpretation by the Uni-G program. The STEMI criteria had a specificity of 97.7% and sensitivity of 27.1% (95% CI, 18.4%-35.8%). The Uni-G STEMI equivalents had a lower sensitivity at 15.4% (95% CI, 8.8%-21.2%), but a higher specificity of 99.4%.

**Table 2:** Comparison to STEMI Criteria.

	AUC	NPV	PPV	Sensitivity	Specificity
**History**	0.717 (0.677-0.757)	0.982 (0.979-0.986)	0.073 (0.031-0.108)	0.103 (0.042-0.151)	0.975 (0.971-0.979)
**History + ECG**	0.880 (0.848-0.917)	0.989 (0.986-0.992)	0.283 (0.218-0.345)	0.436 (0.345-0.522)	0.979 (0.975-0.983)
**History + ECG + POC**	0.890 (0.859-0.927)	0.990 (0.987-0.992)	0.273 (0.211-0.330)	0.479 (0.393-0.566)	0.975 (0.972-0.979)
**History + ECG + hs-cTnT**	0.948 (0.932-0.969)	0.993 (0.991-0.995)	0.326 (0.262-0.386)	0.641 (0.554-0.729)	0.974 (0.970-0.978)
**History + ECG + POC + hs-cTnT**	0.953 (0.938-0.973)	0.992 (0.990-0.995)	0.353 (0.285-0.414)	0.615 (0.527-0.700)	0.978 (0.975-0.982)
STEMI	N/A	0.987 (0.984-0.990)	0.172 (0.117-0.228)	0.271 (0.184-0.358)	0.977 (0.973-0.981)
Uni-G STEMI	N/A	0.985 (0.982-0.988)	0.172 (0.110-0.230)	0.222 (0.141-0.296)	0.979 (0.975-0.983)

AUC, area under the receiver operating characteristic curve; ECG, electrocardiogram; hs-cTnT, high-sensitivity cardiac troponin T; NPV, negative predictive value; POC, point-of-care blood tests; PPV, positive predictive value; STEMI, ST-segment elevation myocardial infarction.

Results of the AI model (bold) using different inputs, compared to the STEMI criteria and the Uni-G STEMI equivalents. Input to the AI model is indicated by the left column. STEMI was evaluated on the validation set excluding left bundle branch block, left ventricular hypertrophy, or ventricular pacing. The AI model and Uni-G STEMI were evaluated on the full validation set. Thresholds for the AI model were chosen such that specificity on the tuning set was the same as the STEMI criteria (97.4%). Numbers in parentheses are 95% CIs, approximated through bootstrapping with *B* = 1000 bootstrap samples. History refers to features derived from historical patient data.

The specificity of the STEMI criteria on the tuning set was 97.4%. To facilitate the comparisons, the decision threshold for the AI model was therefore chosen so that the specificity in the tuning set was 97.4%.

There was a clear improvement in the model predictions when adding the ECG and again when adding hs-cTnT. The benefit of the POC blood samples seemed to be marginal. The AI model using all inputs reached an AUC of 95.3% (95% CI, 93.8%-97.3%) and had a sensitivity of 61.5%, compared with 27.1% for the STEMI criteria at similar specificity, corresponding to a 34.5% increase (95% CI, 22.9%-45.2%). A full comparison showing all possible combinations of input feature groups is reported in Supplementary Appendix 4: Table S7. Results for the AI model evaluated on the cohort excluding patients with LBBB, LVH, and VP are in Supplementary Appendix 4: Table S8.

**Figure 4:**
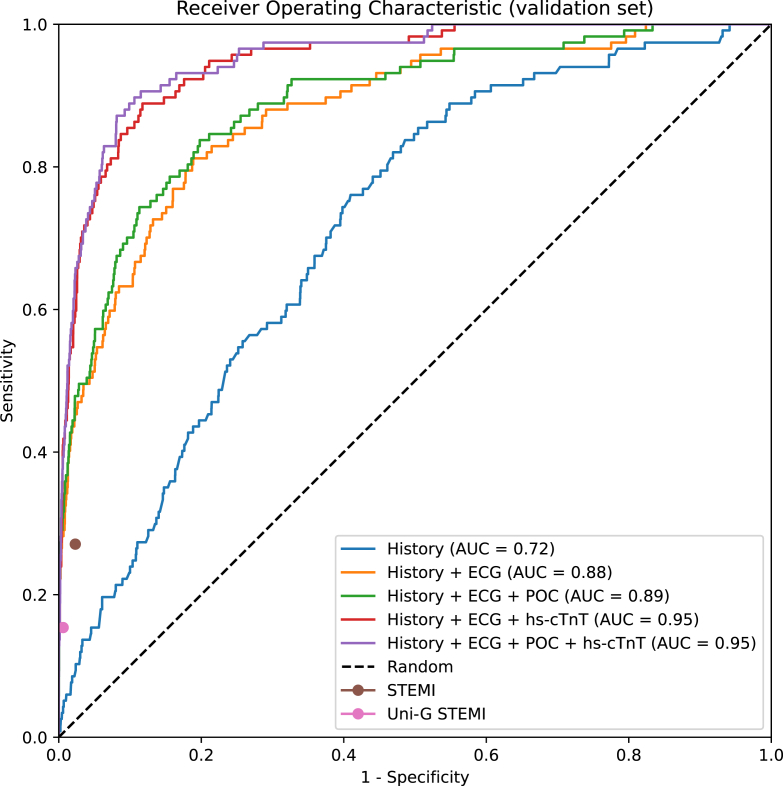
Receiver operating characteristic (ROC) curves for OMI classification. ROC curves for the AI model evaluated on the validation set using different inputs that correspond to when new information becomes available at the emergency department. The positive class is OMI; the negative class is everyone else. Also plotted are the STEMI criteria and the Uni-G algorithm statements corresponding to STEMI. The dashed line corresponds to randomly guessing the outcome. AUC, area under the ROC curve; ECG, electrocardiogram; hs-cTnT, high-sensitivity cardiac troponin T; OMI, occlusion myocardial infarction; POC, point-of-care blood samples; ROC, receiver operating characteristic; STEMI, ST-segment elevation myocardial infarction.

The receiver operating characteristic (ROC) curves are plotted in Figure 4, together with the ROC points of the STEMI criteria and the Uni-G STEMI interpretations. Notably, both the STEMI criteria and the Uni-G STEMI decision points fall under the ROC curves of the AI model, except when the model uses only historical patient data.

All models, including the STEMI criteria and the Uni-G STEMI interpretations, performed better for women than men. For example, the model including all features (history + ECG + POC + hs-cTnT) had an AUC of 97.2% (95% CI, 95.8%-98.8%) for women, compared with 93.8% AUC (95% CI, 91.1%-96.8%) for men. The full results are listed in Supplementary Appendix 3.

### 3.4 Usability in the Context of Clinical Care

The prediction models described in this paper are mainly designed as a foundation for clinical decision support tools. In practice, clinicians must consider additional information not captured by the model. Moreover, potential users must be adequately informed about the strengths and limitations of the model. In the ideal case, such a decision support tool should be seamlessly integrated into the clinical workflow such that manual data entry is entirely avoided as it reduces the effort to use the tool while also decreasing the chance of erroneous usage. To evaluate the impact on patient care, prospective clinical trials will be required.

## 4 Limitations

This study has several limitations. Despite significant efforts to label OMI accurately, it is nevertheless possible that some misclassifications were made. For example, the degree of stenosis and occlusion can change dynamically over time and be affected by treatments with antiplatelet and anticoagulation therapies, making retrospective annotation difficult. Additionally, some OMI cases lacking angiography (*n* = 40) may have been misclassified despite the use of published definitions. However, we believe that including patients without angiography is important for ensuring that the model and validation reflect the entire ED chest-pain population and capture potential cases of missed relevant interventions.

In this study, we used a linear model (logistic regression) for the final classification of OMI, which could be viewed as a limitation. However, the most complex input by far is the raw ECG signal, which is processed by a nonlinear deep-learning model. Initial experiments also indicated that multilayer feed-forward neural networks and random forest models would not perform better than logistic regression on this dataset. Thus, we favored a simple and robust model in the final stage, allowing for a more direct comparison between combinations of input features.

The validation was conducted retrospectively in patients presenting to the ED during 2017-2018, which may limit generalizability. However, given the use of high-sensitivity biomarkers, stable AMI definitions, and unchanged guidelines on acute intervention during this period, we believe the results are still promising. Nevertheless, prospective validation in contemporary cohorts will be required before clinical implementation can be considered.

The study population included ED patients in a setting where obvious prehospital STEMI cases routinely bypassed the ED. Although this reflects the clinical reality in Sweden and elsewhere, the generalizability of the results and the model may be limited in settings without such a bypass system.

We compared the predictions of the AI model with an automatic evaluation of strict ECG criteria for STEMI. It would have been interesting to also compare our model to the physicians’ interpretation of the ECG, as well as their clinical suspicion of OMI based on the available information. Previous studies suggest that physicians are better than the STEMI criteria at detecting OMI,^11^ but results are mixed when comparing with AI models predicting OMI. One study found that their AI model was better than doctors,^15^ and another that ECG experts performed equally well as their AI model.^16^

Despite our efforts to select the most important inputs to the AI model, additional variables might be valuable to consider. In particular, symptom duration is likely to be useful, but this was unfortunately not available in the ESC-TROP database.

## 5 Discussion

In a cohort of 24,511 unselected ED patients with chest pain, we found that 467 (1.9% of the total, 29% of AMI cases) had OMI. Our AI model used the 12-lead ECG in combination with patient history data and initial blood test values to achieve an AUC of 95.3% for predicting OMI in the internal validation (6128 patients). To our knowledge, this represents the highest-performing OMI prediction model published to date. Furthermore, we found that only 5.4% of the identified OMI cases received reperfusion therapy within the maximum 90 minutes recommended by international guidelines,^3^^,^^30^ possibly causing a higher mortality.^1^ Our AI model had more than twice as high sensitivity and PPV as the STEMI criteria at the same specificity and could have helped to identify a majority of the OMI cases, reducing the time to treatment.

We are aware of only 2 published AI-based OMI prediction models, and both are based solely on the ECG. Al-Zaiti et al^15^ developed the ECG-SMART model to predict OMI in patients with chest pain based on ECGs recorded in the ambulance, excluding STEMI cases. Their model achieved an AUC of 91%, with an OMI prevalence of 6.4% in the validation set. Herman et al^16^ created the Queen of Hearts model, which achieved an AUC of 94% for predicting angiographically adjudicated OMI in a mixed cohort of suspected ACS patients, with a validation set OMI prevalence of 22%.

Although our model required the addition of the initial hs-cTnT to achieve a numerically higher AUC (our model had an AUC of 87% when using the ECG alone), it is important to remember that differences between populations can make direct AUC comparisons problematic. For example, our cohort has a substantially lower prevalence of OMI, and the prehospital screening of STEMI patients may have resulted in a higher proportion of complex cases, because the most “obvious” STEMI cases were absent. An evaluation on the same cohort would be required to meaningfully compare our model with those of Al-Zaiti and Herman. In any case, our efforts support the previous conclusion that AI models consistently and considerably outperform traditional STEMI criteria.

We found that OMI was associated with poorer outcomes than NOMI, with a doubled crude 30-day mortality rate, despite OMI patients being younger and having fewer comorbidities. This is in agreement with Khan et al,^7^ who reported a 2-fold increase in mortality among NSTEMI patients with OMI vs NOMI, and highlights the critical need for improved management of this important patient group.

We analyzed the predictive value of the different input components of our model. These analyses suggest that the patient history alone is (unsurprisingly) of limited value. With the ECG available, the benefit of patient history data as well as POC blood tests seems to be marginal. When the first hs-cTnT is included, the predictive value of the ECG is substantially reduced. A combination of all input features seems to yield the best result, although further analyses are needed to clarify whether the added complexity of including patient history data is justified.

Predictions based on hs-cTnT samples would naturally be delayed compared with those based on the ECG alone, and elucidating whether the improvement in performance is worth the wait will require further investigations. However, advancements in POC testing now make it feasible to obtain hs-cTnT within minutes, and possibly already in the ambulance, which would largely ameliorate such concerns.^31^

Despite an AUC over 95%, the low prevalence of OMI (1.9%) presents a practical challenge. Even at a 97.4% specificity and an NPV over 99%, the PPV was only 35%, which may be insufficient for emergent angiography activation. Adjusting the decision threshold would result in higher specificity and PPV, but with reduced sensitivity. A proper cost-benefit analysis would be necessary to properly balance the trade-offs. Regardless of the threshold set, these models serve as decision support tools. The ultimate decision of emergent angiography should be made by physicians, who can consider all available clinical information and patient-specific factors essential for confirming the need for intervention.

In many EDs, ECGs are presented to physicians with interpretations by rule-based algorithms that indicate when STEMI is suspected or present. To compare our model with such a system, we utilized the Uni-G ECG program, which demonstrated a sensitivity for OMI of 15.4% and a specificity of 99.4%. By comparison, Al-Zaiti et al^15^ evaluated a commercial ECG interpretation system (Philips DXL diagnostic algorithm) and found a sensitivity of 79% and specificity of 80% for OMI in patients with chest pain with ECGs from ambulances. In both Al-Zaiti’s and our populations, the findings indicate that AI models outperform rule-based interpretations using STEMI criteria in predicting OMI.

Because the STEMI criteria are undefined for patients with LBBB, LVH, or VP, they were evaluated only in the cohort where those patients were excluded. This of course favored the STEMI criteria, which would otherwise have appeared worse. The AI model did not perform differently when evaluated in the reduced cohort, as shown in Supplementary Appendix 4.

In summary, we developed an AI model that can predict OMI in ED patients with chest pain with an AUC around 95% using data that is commonly available early in the diagnostic process. At a similar specificity (97.4%), the model was more than twice as sensitive for OMI as the standard STEMI criteria (62% vs 27%). Almost a third of all AMI patients were found to have OMI, with a doubled 30-day mortality rate, but only 5.4% of the OMI patients received coronary angiography within the recommended 2 hours. It is possible that many of these patients could have been identified earlier with the support of our AI-based decision tool.

## Author Contributions

**Axel Nyström**: Conceptualization, Methodology, Software, Validation, Formal analysis, Investigation, Data curation, Writing—original draft, Writing—review and editing, Visualization.

**Anders Björkelund**: Conceptualization, Methodology, Validation, Writing—review and editing, Supervision, Project administration.

**Henrik Wagner**: Investigation, Data curation, Writing—review and editing.

**Ulf Ekelund**: Conceptualization, Resources, Writing—review and editing, and Funding acquisition.

**Mattias Ohlsson**: Conceptualization, Methodology, Resources, Writing—review and editing, Supervision, Project administration.

**Jonas Björk**: Conceptualization, Resources, Writing—review and editing, Funding acquisition.

**Arash Mokhtari**: Data curation, Writing—review and editing.

**Jakob Lundager Forberg**: Conceptualization, Methodology, Validation, Investigation, Resources, Data curation, Writing—original draft, Writing—review and editing, Visualization, Supervision, Project administration.

## Funding and Support

This study received funding from the 10.13039/501100004359Swedish Research Council (https://www.vr.se/english.html, VR; grant no. 2019-00198, awarded to JB), the 10.13039/501100003793Swedish Heart-Lung Foundation (https://www.hjart-lungfonden.se/, grant no. 2018 0173, awarded to UE), and Sweden’s innovation agency Vinnova (https://www.vinnova.se/en/, grant no. 2018-0192, grant awarded to JB). The funders had no role in study design, data collection and analysis, decision to publish, or preparation of the manuscript.

## Data Sharing Statement

This study is based on sensitive individual-level, pseudonymized, healthcare data from the ESC-TROP study (https://doi.org/10.1159/000509390) and cannot be made publicly available for ethical and legal reasons (c.f. the Public Access to Information and Secrecy Act, as well as the General Data Protection Regulation). However, we welcome initiatives on international collaborative projects. Anonymized parts of the database can be made available for researchers upon reasonable request, although this may require additional ethical permits. Inquiries can be sent to Ulf Ekelund (ulf.ekelund@med.lu.se) at Lund University or to Clinical Studies Sweden, Forum South (halsodata.sus@skane.se). The code used in this study is available at https://www.github.com/Tipulidae/mim.

## Conflict of Interest

All authors have affirmed they have no conflicting interests to declare.

## Supplemental material

Supplementaty material
