# Supplementary material for: Predicting Occlusion Myocardial Infarctions in the Emergency Department Using Artificial Intelligence
Source: J Am Coll Emerg Physicians Open. 2026 Jan 9;7(1):100299. doi: 10.1016/j.acepjo.2025.100299 (PMC12818230; doi:10.1016/j.acepjo.2025.100299)
Supplement: Supplementaty material [file mmc1.pdf]

Supplementary Appendix 1. Feature groups

Feature group	Feature	Datatype	ICD-10 / ATC / KVÅ codes
History	Age	Float (years)	
	Female sex	Boolean	
	Male sex	Boolean	
	Ischaemic heart diseases	Boolean	I20-I25
	Acute myocardial infarction	Boolean	I21, I22
	Angina pectoris	Boolean	I20
	Unstable angina	Boolean	I200
	Heart failure	Boolean	I50
	Diabetes	Boolean	E10, E11
	Hypertension	Boolean	I10-I13, I15
	Hypercholesterolemia	Boolean	E78
	Pulmonary embolism	Boolean	I26
	Cerebrovascular diseases	Boolean	G45, I63
	Chronic obstructive pulmonary disease	Boolean	J44
	Renal failure	Boolean	N18, N19, I13
	Arteries/arterioles/capillaries diseases	Boolean	I70, I71, I74
	Depression	Boolean	F32, F33
	Prior CABG	Boolean	FNA, FNB, FNC, FND, FNE, FNF
	Prior PCI	Boolean	FNG
	Antithrombotics	Boolean	B01AC
	Statin	Boolean	C10AA
	Other lipid-lowering medication	Boolean	C10 (excluding C10AA)
	Antihypertensive medication	Boolean	C02, C08C, C08GA, C09
	Beta blockers	Boolean	C07
	Anticoagulants	Boolean	B01AA
	Insulin	Boolean	A10A
	Other antidiabetics	Boolean	A10B, A10X
	Diuretics	Boolean	C03C, C03D, C03E
	Thiazide	Boolean	C03A
	Antiarrhythmic drugs	Boolean	C01
	ACE/AII-antagonists	Boolean	C09
	Calcium Blocker	Boolean	C08
POC	Creatinine	Float (µmol/L)	
	Hemoglobin	Float (g/L)	
	Glucose	Float (mmol/L)	
	Indicator variable for missing samples	Boolean	
hs-cTnT	hs-cTnT	Float (ng/L)	
	log(hs-cTnT)	Float	
	Time until hs-cTnT	Float (hours)	
	Indicator variable for missing hs-cTnT	Boolean	

Table S1: Feature groups. The diagnoses are based on ICD-10 codes from the Swedish patient register going back 5 years prior to the index visit. The corresponding feature was coded as True if any of the listed diseases were diagnosed at any point in the 5-year history, otherwise False. Similarly for the medications, except the codes are ATC-codes and they only go back 3 years. The medical treatments are based on "KVÅ–codes", also from the Swedish patient register. CABG = Coronary artery bypass graft surgery, PCI = Percutaneous coronary intervention, hs-cTnT = high-sensitivity cardiac troponin T.

1.1. *Blood sample analyses*

Glucose, hemoglobin and creatinine were measured with either Radiometer ABL 800 flex Blood Gas Analyser or Cobas 6000 (Roche Diagnostics) for all study sites. The hs-cTnT samples were collected in lithium heparin tubes and analyzed with Cobas e602 (Roche Diagnostics). The assay has a limit of detection of 5 ng/L and a limit of blank of 3 ng/L. The 99th percentile cut-off point is 14 ng/L and the coefficient of variation is 10% at 13 ng/L.

Supplementary Appendix 2. Machine learning details

2.1. *Transfer learning of the ECG model*

In order to improve the performance of the ECG model we employed a form of transfer learning, in which the model was first *pre-trained* to predict age, and subsequently *fine-tuned* to predict OMI. Our previous research showed that such a strategy can substantially boost the performance when the downstream task is to predict AMI [1]. For the pre-training step, we used a collection of 800 k ECGs from the Skåne Emergency Medicine (SEM) database [2], all from ED patients without any registered chest-pain episodes. It should be noted that our use of transfer learning in this context was purely a means to obtain better OMI predictions; the goal was not to predict age as such.

We used a 1-dimensional ResNet architecture, selecting model parameters through a multi-step process. In the first step, we used random search over a space of possible model variations, where in each iteration a model was trained from scratch on data from the SEM dataset to predict patient age from the raw ECG signal. In the second step, we used random search to select parameters for fine-tuning. In each iteration, a pre-trained model from the first step was selected at random from the top 50 best models with respect to mean absolute error on the pre-training task. In order to reduce overfitting on the OMI target, the fine-tuning task for the second step of the model selection was to predict AMI among chest-pain patients from the SEM database (44 370 visits) – a similar task on a similar dataset. We used the same data splits as described in Nyström *et al.* [1]. The final ECG model was created by using the best performing hyper-parameters from the second round of random search, fine-tuning on the OMI task using the ESC-TROP database.

2.2. *Random search for pre-training*

The model architecture for ECG pre-training was selected through random search with 500 iterations. The hyper-parameter search space is shown in Table S2. The training target was age (regression), with L1 loss and Adam optimizer. Learning-rate followed a one-cycle schedule [3] with 10 epoch linear warmup to the randomly selected target, followed by a factor 100 cosine decay over 90 epochs. Early-stopping was used with a patience of 15 epochs, monitoring L1 loss on the tuning set. Batch size was 512, and the total training size was 794 k ECGs from the SEM cohort.

**Hyper-parameter**	**Search space**
Architecture	ResNet 18, ResNet 34, ResNet 50, ResNet 101, ResNet 152, ResNeXt 50 (32x4), ResNeXt 101 (32x8), ResNeXt 101 (64x4)
Dropout	0.2, 0.3, 0.4, 0.5, 0.6, 0.7, 0.8
Initial kernel-size	5, 9, 13, 17, 21, 25
Initial stride	2, 4, 6, 8, 10
Squeeze-Excitation	True, False
Squeeze-Excitation reduction ratio	4, 8, 16, 32
Learning-rate warmup target	1e-5, 3e-5, 1e-4, 3e-4, 1e-3, 3e-3

Table S2: Hyper-parameter search space for pre-training random search. In each iteration, the value of each parameter (left column) is selected independently at random from the search space (right column).

2.3. *Random search for fine-tuning*

The settings for fine-tuning the ECG were also decided through random search with 500 iterations, using the hyper-parameter search space shown in Table S3. Each iteration, a randomly chosen pre-trained ECG model was selected, and the final layer was replaced by a dropout layer and a fully connected layer. The model was then trained for 40 epochs with all layers frozen except for the final layer. The learning rate followed a one-cycle schedule with a 10 epoch linear warmup to a randomly selected target, followed by a cosine decay of a factor 100 over 20 epochs. After 40 epochs, all layers were unfrozen and training continued for an additional 160 epochs. The prediction target was AMI within 30 days with binary cross-entropy as loss function and Adam as optimizer. The training data was chest-pain patients in the SEM cohort (24 916 ECGs). Early stopping was used, monitoring AUC on the tuning set with a patience of 15 epochs.

**Hyper-parameter**	**Search space**
Pre-trained model	Top-50 models (w.r.t. tuning loss) from 500 pre-trained models (previous random search)
Dropout	0.2, 0.3, 0.4, 0.5, 0.6, 0.7, 0.8
Fully connected size	50, 100, 200
Learning-rate warmup target	1e-4, 3e-4, 1e-3, 3e-3, 1e-2, 3e-2

Table S3: Hyper-parameter search space for fine-tuning random search. In each iteration, the value of each parameter (left column) is selected independently at random from the search space (right column).

2.4. *Final ResNet parameters*

The final ECG model was a 1-dimensional version of the ResNeXt-101 (32x8) architecture [4]. The initial kernel size was 17, with subsequent kernel size 5 and a stride of 2. The model used a Squeeze and Excitation (SE) [5] module with a reduction ratio of 16 in each bottleneck block. The pre-training phase used a dropout of 0.2 and a batch-size of 512, using L1-loss and the Adam optimizer. The learning-rate followed a one-cycle schedule with a linear warmup from 1e-6 to 1e-3, followed by a cosine decay to 1e-5 over 90 epochs. Early stopping was used with a patience of 15 epochs monitoring the L1-loss on the tuning set.

In the fine-tuning step, the model weights from the best performing epoch with respect to tuning loss were loaded, and the output layer was replaced by a dropout (0.4) followed by a fully-connected layer of size 50 with random (kaiming) initialization. The model was then trained (target OMI, binary cross-entropy loss and Adam optimizer) for 40 epochs with the full network frozen, except for the final layer. The learning-rate was once again a one-cycle schedule, with peak 3e-4 after 10 epochs and cosine decay to 3e-6 over 20 epochs. All weights were then unfrozen and training resumed for an additional 280 epochs with a fixed learning rate of 3e-6 and a batch size of 256.

Supplementary Appendix 3.

Tables S4 and S5 show the results stratified on sex. All models, including the STEMI criteria and Uni-G STEMI, performed better for women than for men. It should be noted that the OMI outcome was more than twice as common among men (2.5% for males, compared to 1.2% for females, in the test group). Although this observation might be explained by differences between the sexes in the pathophysiology of cardiovascular disease and OMI, we cannot rule out systematic biases in how AMI is experienced, diagnosed, and treated. It is plausible that our dataset and annotation contain a substantial number of false negative OMI cases among female patients, and that those who were found to have OMI were primarily the more obvious cases. Other potential explanations and confounders include differences in behavior, lifestyle, age, co-morbidities, and other sociodemographic parameters. More research would be required to better understand this effect.

	**AUC**	**NPV**	**PPV**	**Sensitivity**	**Specificity**
**History**	0.664 (0.610–0.722)	0.977 (0.972–0.983)	0.073 (0.031–0.110)	0.150 (0.063–0.226)	0.951 (0.943–0.958)
**History + ECG**	0.864 (0.823–0.907)	0.986 (0.982–0.990)	0.270 (0.197–0.343)	0.463 (0.359–0.568)	0.968 (0.961–0.974)
**History + ECG + POC**	0.873 (0.835–0.915)	0.987 (0.983–0.991)	0.270 (0.199–0.339)	0.500 (0.389–0.617)	0.965 (0.958–0.972)
**History + ECG + hs-cTnT**	0.933 (0.905–0.964)	0.992 (0.989–0.995)	0.331 (0.257–0.402)	0.688 (0.587–0.789)	0.964 (0.957–0.970)
**History + ECG + POC + hs-cTnT**	0.938 (0.911–0.968)	0.990 (0.987–0.994)	0.359 (0.282–0.434)	0.637 (0.528–0.742)	0.970 (0.964–0.976)
STEMI	N/A	0.981 (0.976–0.986)	0.157 (0.085–0.216)	0.257 (0.146–0.350)	0.965 (0.958–0.972)
Uni-G STEMI	N/A	0.980 (0.975–0.985)	0.157 (0.092–0.216)	0.237 (0.142–0.322)	0.967 (0.961–0.973)

Table S4: Results stratified on males.

	**AUC**	**NPV**	**PPV**	**Sensitivity**	**Specificity**
**History**	0.744 (0.668–0.828)	0.988 (0.984–0.992)	nan (nan–nan)	0.000 (0.000–0.000)	1.000 (1.000–1.000)
**History + ECG**	0.885 (0.827–0.954)	0.992 (0.989–0.996)	0.326 (0.179–0.461)	0.378 (0.227–0.526)	0.990 (0.987–0.994)
**History + ECG + POC**	0.895 (0.838–0.964)	0.993 (0.990–0.996)	0.281 (0.161–0.392)	0.432 (0.258–0.586)	0.986 (0.982–0.990)
**History + ECG + hs-cTnT**	0.968 (0.953–0.987)	0.994 (0.991–0.997)	0.302 (0.179–0.416)	0.514 (0.360–0.682)	0.985 (0.981–0.989)
**History + ECG + POC + hs-cTnT**	0.972 (0.958–0.988)	0.994 (0.992–0.997)	0.339 (0.210–0.454)	0.568 (0.411–0.726)	0.986 (0.982–0.990)
STEMI	N/A	0.994 (0.992–0.997)	0.233 (0.055–0.371)	0.318 (0.103–0.494)	0.991 (0.988–0.994)
Uni-G STEMI	N/A	0.990 (0.986–0.994)	0.233 (0.080–0.376)	0.189 (0.055–0.304)	0.992 (0.989–0.995)

Table S5: Results stratified on females.

	Train	Tune	Validation
**Patients, n**	12255	6128	6128
**Female, n (%)**	5935 (48.4)	2896 (47.3)	2971 (48.5)
**Age, years (std)**	60 (18.6)	59 (19.0)	59 (18.8)
**AMI, n (%)**	801 (6.5)	405 (6.6)	383 (6.2)
**OMI, n (%)**	233 (1.9)	117 (1.9)	117 (1.9)
**NOMI, n (%)**	568 (4.6)	288 (4.7)	266 (4.3)
**STEMI criteria, n (%)**	293 (2.4)	169 (2.8)	151 (2.5)
**Ischaemic heart diseases, n (%)**	1797 (14.7)	908 (14.8)	919 (15.0)
**Acute myocardial infarction, n (%)**	742 (6.1)	356 (5.8)	393 (6.4)
**Angina pectoris, n (%)**	792 (6.5)	401 (6.5)	394 (6.4)
**Unstable angina, n (%)**	269 (2.2)	144 (2.3)	133 (2.2)
**Heart failure, n (%)**	740 (6.0)	358 (5.8)	358 (5.8)
**Diabetes, n (%)**	1054 (8.6)	527 (8.6)	505 (8.2)
**Hypertension, n (%)**	2515 (20.5)	1242 (20.3)	1258 (20.5)
**Pulmonary embolism, n (%)**	155 (1.3)	79 (1.3)	68 (1.1)
**Cerebrovascular diseases, n (%)**	435 (3.5)	223 (3.6)	232 (3.8)
**Chronic obstructive pulmonary disease, n (%)**	443 (3.6)	250 (4.1)	232 (3.8)
**Prior CABG, n (%)**	128 (1.0)	63 (1.0)	61 (1.0)
**Prior PCI, n (%)**	687 (5.6)	352 (5.7)	378 (6.2)
**Antithrombotics, n (%)**	2837 (23.1)	1472 (24.0)	1412 (23.0)
**Statin, n (%)**	3325 (27.1)	1670 (27.3)	1690 (27.6)
**Other lipid-lowering medication, n (%)**	323 (2.6)	159 (2.6)	170 (2.8)
**Antihypertensive medication, n (%)**	4458 (36.4)	2262 (36.9)	2248 (36.7)
**Beta blockers, n (%)**	3720 (30.4)	1820 (29.7)	1833 (29.9)
**Anticoagulants, n (%)**	657 (5.4)	327 (5.3)	337 (5.5)
**Insulin, n (%)**	698 (5.7)	354 (5.8)	341 (5.6)
**Other antidiabetics, n (%)**	1217 (9.9)	604 (9.9)	597 (9.7)
**Diuretics, n (%)**	1803 (14.7)	923 (15.1)	895 (14.6)
**Thiazide, n (%)**	539 (4.4)	304 (5.0)	272 (4.4)
**ACE/AII-antagonists, n (%)**	3792 (30.9)	1932 (31.5)	1901 (31.0)
**Initial hs-cTnT (ng/L), median (IQR)**	7 (4 - 15)	7 (4 - 15)	6 (4 - 15)
**Initial lactate (mmol/L), median (IQR)**	1.2 (1.0 - 1.6)	1.2 (1.0 - 1.6)	1.2 (0.9 - 1.6)
**30 day mortality, n (%)**	146 (1.2)	66 (1.1)	74 (1.2)
**180 day mortality, n (%)**	357 (2.9)	169 (2.8)	186 (3.0)
**1 year mortality, n (%)**	507 (4.1)	250 (4.1)	267 (4.4)
**Hours until angiography, median (IQR)**	29 (16 - 64)	29 (18 - 54)	28 (16 - 56)
**Angiography within 90 min, n (%)**	15 (0.1)	7 (0.1)	10 (0.2)
**Angiography within 3 h, n (%)**	87 (0.7)	51 (0.8)	37 (0.6)
**Angiography within 6 h, n (%)**	157 (1.3)	85 (1.4)	79 (1.3)

Table S6: Patient characteristics, data splits. Disease history covers 5 years, medication history 3 years. STEMI criteria evaluated on the index ECG, treating left bundle branch block, left ventricular hypertrophy and ventricular pacing as STEMI negative. See Appendix 1 for ICD10-codes and ATC-codes corresponding to disease and medication history. IQR = Interquartile range, CABG = Coronary Artery Bypass Graft surgery, PCI = Percutaneous Coronary Intervention. Data split is random, but stratified on outcome (OMI), to maintain the same number of OMI patients in each split.

Supplementary Appendix 4. Additional results

History	ECG	POC	hs-cTnT	AUC	NPV	PPV	Sens	Spec
X				0.717 (0.677–0.757)	0.982 (0.979–0.986)	0.073 (0.031–0.108)	0.103 (0.042–0.151)	0.975 (0.971–0.979)
	X			0.872 (0.840–0.906)	0.988 (0.985–0.990)	0.235 (0.168–0.292)	0.376 (0.287–0.459)	0.976 (0.973–0.980)
X	X			0.880 (0.848–0.917)	0.989 (0.986–0.992)	0.283 (0.218–0.345)	0.436 (0.345–0.522)	0.979 (0.975–0.983)
		X		0.751 (0.708–0.798)	0.983 (0.980–0.986)	0.097 (0.055–0.140)	0.137 (0.080–0.194)	0.975 (0.971–0.979)
X		X		0.800 (0.760–0.842)	0.984 (0.981–0.987)	0.139 (0.084–0.184)	0.197 (0.124–0.259)	0.976 (0.972–0.980)
	X	X		0.881 (0.850–0.914)	0.988 (0.986–0.991)	0.241 (0.180–0.299)	0.410 (0.324–0.501)	0.975 (0.971–0.979)
X	X	X		0.891 (0.861–0.928)	0.990 (0.987–0.992)	0.272 (0.213–0.328)	0.479 (0.393–0.566)	0.975 (0.971–0.979)
			X	0.920 (0.898–0.944)	0.990 (0.988–0.993)	0.335 (0.263–0.403)	0.513 (0.419–0.598)	0.980 (0.977–0.984)
X			X	0.936 (0.918–0.958)	0.992 (0.990–0.994)	0.316 (0.254–0.378)	0.581 (0.496–0.668)	0.976 (0.972–0.979)
	X		X	0.943 (0.927–0.963)	0.992 (0.990–0.994)	0.308 (0.246–0.367)	0.581 (0.496–0.667)	0.975 (0.971–0.979)
X	X		X	0.948 (0.932–0.969)	0.993 (0.991–0.995)	0.326 (0.262–0.386)	0.641 (0.554–0.729)	0.974 (0.970–0.978)
		X	X	0.935 (0.916–0.958)	0.992 (0.990–0.994)	0.300 (0.242–0.359)	0.590 (0.503–0.683)	0.973 (0.969–0.977)
X		X	X	0.945 (0.927–0.965)	0.992 (0.990–0.994)	0.337 (0.275–0.400)	0.598 (0.509–0.688)	0.977 (0.973–0.981)
	X	X	X	0.948 (0.932–0.968)	0.992 (0.990–0.995)	0.305 (0.247–0.360)	0.615 (0.531–0.699)	0.973 (0.969–0.977)
X	X	X	X	0.953 (0.937–0.973)	0.993 (0.990–0.995)	0.354 (0.289–0.416)	0.624 (0.532–0.709)	0.978 (0.974–0.982)

Table S7: AI validation performance for different combinations of input features, as indicated by the Xs. Thresholds were chosen such that specificity on the tuning set was 97.5%. Numbers in parenthesis are 95% confidence intervals, approximated through bootstrapping with $B = 1000$ bootstrap samples. History refers to features derived from historical patient data. POC = point-of-care blood tests, hs-cTnT = high-sensitivity cardiac troponin T, NPV = negative predictive value, PPV = positive predictive value, AUC = Area under the receiver operating characteristic.

	AUC	NPV	PPV	Sensitivity	Specificity
**History**	0.728 (0.684–0.776)	0.984 (0.981–0.987)	0.077 (0.033–0.117)	0.115 (0.052–0.174)	0.976 (0.971–0.980)
**History + ECG**	0.880 (0.844–0.920)	0.991 (0.988–0.993)	0.301 (0.220–0.373)	0.479 (0.376–0.577)	0.980 (0.976–0.984)
**History + ECG + POC**	0.887 (0.853–0.926)	0.991 (0.989–0.994)	0.285 (0.210–0.348)	0.510 (0.412–0.611)	0.977 (0.973–0.981)
**History + ECG + POC + hs-cTnT**	0.953 (0.935–0.974)	0.993 (0.991–0.996)	0.361 (0.287–0.430)	0.635 (0.543–0.732)	0.980 (0.976–0.984)
STEMI	N/A	0.987 (0.984–0.990)	0.172 (0.114–0.230)	0.271 (0.181–0.359)	0.977 (0.973–0.981)
Uni-G STEMI	N/A	0.985 (0.982–0.989)	0.300 (0.156–0.421)	0.156 (0.078–0.229)	0.994 (0.991–0.996)

Table S8: Results of AI model (bold) on the validation set excluding left bundle branch block, left ventricular hypertrophy, and ventricular pacing. Input to the AI model is indicated by the left column. Thresholds for the AI model were chosen such that specificity on the tuning set was the same as the STEMI-criteria (97.4%). Numbers in parenthesis are 95% confidence intervals, approximated through bootstrapping with $B = 1000$ bootstrap samples. History refers to features derived from historical patient data. POC = point-of-care blood tests, hs-cTnT = high-sensitivity cardiac troponin T, NPV = negative predictive value, PPV = positive predictive value, AUC = Area under the receiver operating characteristic.

References

[1] A. Nyström, A. Björkelund, M. Ohlsson, J. Björk, U. Ekelund, J. Lundager Forberg, Transfer learning for predicting acute myocardial infarction using electrocardiograms, PLOS Digital Health 4 (2025) 1–19. doi:10.1371/journal.pdig.0001058.

[2] U. Ekelund, B. Ohlsson, O. Melander, J. Björk, M. Ohlsson, J. Lundager Forberg, P. Olsson de Capretz, A. Nyström, A. Björkelund, The Skåne Emergency Medicine (SEM) cohort, Scandinavian Journal of Trauma, Resuscitation and Emergency Medicine 32 (2024) 37. doi:10.1186/s13049-024-01206-0.

[3] L. N. Smith, N. Topin, Super-convergence: Very fast training of neural networks using large learning rates, 2018. URL: https://arxiv.org/abs/1708.07120. arXiv:1708.07120.

[4] S. Xie, R. Girshick, P. Dollár, Z. Tu, K. He, Aggregated residual transformations for deep neural networks, 2017. URL: https://arxiv.org/abs/1611.05431. arXiv:1611.05431.

[5] J. Hu, L. Shen, G. Sun, Squeeze-and-excitation networks, in: Proceedings of the IEEE Conference on Computer Vision and Pattern Recognition (CVPR), 2018. doi:10.48550/arXiv.1709.01507.
